# Case Report: Indocyanine green fluorescence imaging in complex focal nodular hyperplasia resection: report of two cases

**DOI:** 10.3389/fmed.2026.1869590

**Published:** 2026-06-15

**Authors:** Fangkai Du, Xie Song, Wentao Wang, Huizhong Shi, Zhengjian Wang, Chaoqun Ma, Qingqiang Ni, Shunzhen Zheng, Hong Chang

**Affiliations:** The Department of Hepatobiliary Surgery, Shandong Provincial Hospital Affiliated to Shandong First Medical University, Jinan, Shandong, China

**Keywords:** fluorescence-guided surgery, focal nodular hyperplasia, indocyanine green, laparoscopic liver resection, punctate hyperfluorescent foci

## Abstract

This report describes indocyanine green (ICG) fluorescence guidance combined with intraoperative ultrasonography (IOUS) for laparoscopic parenchyma-sparing excision of selected perivascular focal nodular hyperplasia (FNH). In two patients with segment IV lesions abutting the portal vein, preoperative ICG administration (0.5 mg/kg, 24 h prior) enabled real-time intraoperative fluorescence imaging integrated with ultrasonography. The dual-modality navigation provided clear visual contrast, facilitating precise lesion delineation and margin assessment. Both patients underwent successful wedge excision with negative surgical margins, with estimated blood loss of 50–20 mL, respectively. Incidental punctate hyperfluorescent foci were observed on the liver surface; one excised focus showed benign hyperplastic changes on histology, while the clinical significance of the remaining foci remains uncertain. These cases suggest that ICG fluorescence may assist laparoscopic parenchyma-sparing excision of selected perivascular FNH lesions, though further studies are needed to define its reproducibility, limitations, and clinical value.

## Introduction

Focal nodular hyperplasia (FNH) is among the most prevalent benign liver lesions and is typically managed conservatively, often regarded as a “do-not-touch” entity when patients are asymptomatic and the diagnosis is secure (1, 2). Characteristic imaging findings usually justify a non-invasive approach; however, diagnostic uncertainty may persist in atypical presentations, including small lesions without a classic central scar or lesions located in perivascular regions, where differentiation from hepatocellular adenoma (HCA) or well-differentiated hepatocellular carcinoma (HCC) may be difficult (3–5). In selected cases, resection may therefore be considered to obtain a definitive histopathological diagnosis, address symptoms when present, or manage documented progression or persistent diagnostic doubt (1, 2). In the present report, complex perivascular FNH refers to lesions with either (1) atypical radiological features (e.g., absence of central scar, non-homogeneous enhancement) or (2) location within 5 mm of first- or second-order portal pedicles or within 5 mm of the main trunks of the left, middle, or right hepatic veins, where standard resection carries an elevated risk of vascular injury or may compromise a parenchyma-sparing strategy. Indocyanine green (ICG) fluorescence imaging has been used as an intraoperative adjunct in hepatobiliary surgery for lesion localization, anatomical liver segmentation, and biliary mapping (6–8). Its combination with laparoscopic ultrasound has also been proposed to improve intraoperative navigation during minimally invasive liver resection (3). Although ICG-guided resection of FNH has previously been reported, including a robotic series of 23 cases (4), limited information is available regarding its use in laparoscopic parenchyma-sparing excision of radiologically indeterminate FNH located immediately adjacent to major portal venous branches. The present report therefore focuses on the technical integration of ICG fluorescence and IOUS for superficial lesion delineation, deep margin assessment, and vascular preservation in this specific perivascular setting.

## Case description

### Case 1

A 50-year-old woman with no relevant medical history, including no history of viral hepatitis, alcohol use, or oral contraceptive use, was found to have an incidental left hepatic lesion on abdominal ultrasound. Contrast-enhanced CT revealed a 3.3 × 2.1 cm hypervascular nodule in the left medial segment (Figure 1). Although the lesion showed relative hyperdensity on delayed phases, the absence of a classic central scar, its small size, and its close proximity to a segmental vessel rendered the imaging features atypical and precluded a definitive diagnosis of FNH. Preoperative laboratory evaluation showed preserved liver function, including alanine aminotransferase, aspartate aminotransferase, total bilirubin, albumin, and international normalized ratio. Routine biochemical tests, coagulation profile, and tumor markers were within normal limits. Serum alpha-fetoprotein (AFP), carcinoembryonic antigen (CEA), and carbohydrate antigen 19-9 (CA19-9) levels were not elevated, and serological tests for hepatitis B and C were negative. Serial imaging showed interval growth from 2.5 × 1.6 cm to 3.3 × 2.1 cm over 6 months. This documented growth, together with atypical imaging features and proximity to the sagittal portion of the left portal vein, led to persistent diagnostic uncertainty (Figure 1). Hepatobiliary phase magnetic resonance imaging (MRI) was not performed before surgery, which is a limitation of the preoperative diagnostic workup. After preoperative intravenous ICG administration 24 h before surgery, intraoperative fluorescence imaging demonstrated a sharply demarcated hyperfluorescent lesion (Figure 2). This finding, together with IOUS, assisted parenchyma-sparing wedge excision. Operative time was 125 min, and estimated blood loss was 50 mL. Several incidental surface punctate hyperfluorescent micro-foci were observed but were not resected (Figure 2). Histopathology confirmed FNH with a negative surgical margin (minimum margin, 5 mm). The postoperative course was uneventful, with no bile leakage, hemorrhage, infection, or liver failure. The abdominal drain was removed on postoperative day 4, and the patient was discharged on the same day. The highest postoperative Clavien-Dindo grade was 0, and liver function tests normalized by postoperative day 2. At 8-month follow-up, the patient was asymptomatic and showed no imaging evidence of residual or recurrent lesion.

**Figure 1:**
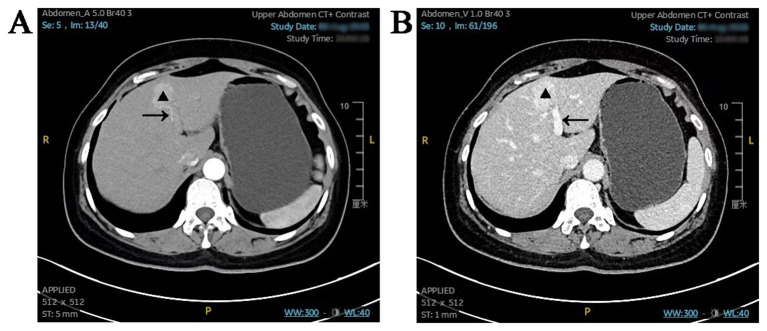
Preoperative contrast-enhanced computed tomography (CT) of focal nodular hyperplasia (FNH) in the left medial hepatic segment (Case 1); **(A)** Arterial phase CT showed a plaque-like enhancing lesion in the left medial segment, adjacent to the falciform ligament and the left hepatic artery **(B)** Portal venous phase CT demonstrated a slightly hyperdense lesion in the same region, abutting the sagittal portion of the left portal vein (▴: FNH, → : Left branch of the hepatic artery, ←: The sagittal part of the left branch of the portal vein).

**Figure 2:**
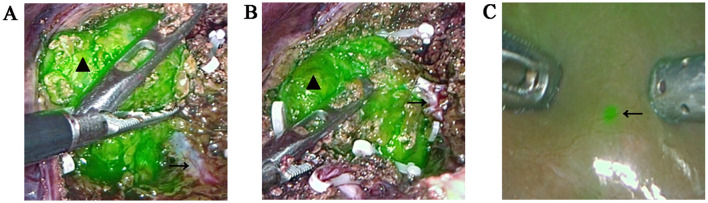
Intraoperative indocyanine green (ICG) fluorescence imaging during laparoscopic resection of FNH in the left medial segment (Case 1); **(A, B)** ICG fluorescence demonstrated a well-demarcated, intensely fluorescent lesion that sharply contrasted with the surrounding dark liver parenchyma and lay adjacent to the sagittal portion of the left portal vein **(C)** Fluorescence laparoscopy revealed multiple punctate hyperfluorescent foci on the liver surface, one of which was shown here [▴: FNH, → : The branch of the portal vein (sagittal portion), ←: Punctate hyperfluorescent focus].

### Case 2

A 51-year-old woman with no history of viral hepatitis, alcohol use, or oral contraceptive use underwent screening abdominal ultrasound, which detected a 1.0 cm lesion in the left medial segment. Subsequent MRI demonstrated a round lesion with diffusion restriction, avid arterial enhancement, and slightly high signal on portal and delayed phases (Figure 3). Liver function tests, routine biochemical tests, and coagulation parameters were normal. AFP, CEA, and CA19-9 levels were not elevated, and hepatitis B and C serologies were negative. Although the lesion was small, its arterial hyperenhancement, diffusion restriction, and location at the portal venous confluence raised a differential diagnosis that included atypical FNH, hepatocellular adenoma, and early hepatocellular carcinoma (Figure 3). Because imaging findings were not considered fully diagnostic and the lesion was in close proximity to major portal venous branches, the case was reviewed by a multidisciplinary team. After discussion of surveillance, biopsy, and surgical excision, laparoscopic parenchyma-sparing resection was selected because of persistent diagnostic uncertainty, technical feasibility, and the patient's preference after informed consent. Intraoperative ICG fluorescence imaging demonstrated a sharply defined hyperfluorescent nodule, which, combined with IOUS, assisted parenchyma-sparing excision (Figure 4). Operative time was 220 min, and estimated blood loss was 20 mL. An incidental 0.5 cm punctate hyperfluorescent focus was excised and showed benign hyperplastic changes (Figure 4). Final pathology confirmed FNH with a negative surgical margin (minimum margin, 5 mm). The postoperative course was uneventful, with no bile leakage, hemorrhage, infection, or liver failure. On postoperative day 5, the abdominal drain was removed and the patient was discharged. The highest postoperative Clavien-Dindo grade was 0, and liver function tests normalized by postoperative day 3. At 9-month follow-up, the patient was asymptomatic and showed no imaging evidence of residual or recurrent lesion.

**Figure 3:**
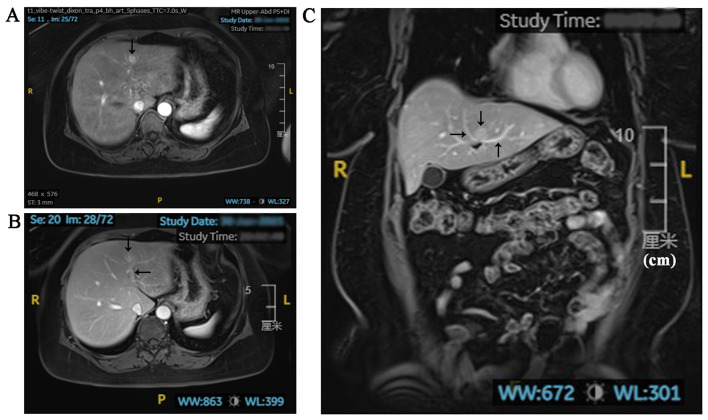
Preoperative contrast-enhanced MRI of FNH adjacent to the sagittal portion of the portal vein (Case 2); **(A)** Arterial-phase image showed a round, intensely enhancing lesion adjacent to two vessels in the sagittal portal venous territory **(B)** Portal-venous-phase image demonstrated a round, mildly hyperintense lesion situated at the confluence of the portal vein branches supplying the left medial and left lateral lobes **(C)** Sagittal reformatted image revealed the lesion immediately superior to the portal vein bifurcation, in close contact with the surrounding vessels (↓: FNH, ←: The sagittal part of the left branch of the portal vein, → : Left medial branch of the portal vein, ↑: Left lateral branch of the portal vein).

**Figure 4:**
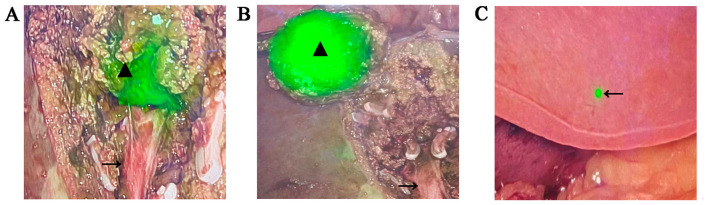
Intraoperative ICG fluorescence imaging during laparoscopic resection of FNH (Case 2); **(A)** Fluorescence laparoscopy revealed a strongly hyperfluorescent lesion that sharply contrasted with the dark surrounding liver parenchyma and directly abutted a branch of the portal vein along its inferior aspect **(B)** Following resection, the excised specimen showed intense fluorescence, confirming clear separation from adjacent portal venous branches **(C)** A separate punctate hyperfluorescent focus on the left diaphragmatic liver surface was identified and excised; histopathology confirmed benign nodular hyperplastic change [▴: FNH, → : The branch of the portal vein (sagittal portion), ←: Punctate hyperfluorescent focus].

Written informed consent was obtained from both patients for publication of their clinical details and accompanying images. Patient perspectives were obtained during postoperative follow-up; both patients reported satisfaction with the treatment outcome and postoperative recovery, and both stated that definitive pathological diagnosis helped relieve anxiety related to the indeterminate liver lesions. A comparative summary of the two cases is provided in Table 1, and the clinical timelines are summarized in Table 2.

**Table 1:** Comparative summary of the two cases.

Variable	Case 1	Case 2
Age and sex	50-year-old woman	51-year-old woman
Relevant medical history	No history of viral hepatitis, alcohol use, or oral contraceptive use	No history of viral hepatitis, alcohol use, or oral contraceptive use
Mode of detection	Incidental lesion detected by abdominal ultrasound	Lesion detected by screening abdominal ultrasound
Preoperative laboratory evaluation	Liver function tests, routine biochemical tests, coagulation profile, tumor markers, and viral hepatitis panel were within normal limits	Liver function tests, routine biochemical tests, coagulation profile, tumor markers, and viral hepatitis panel were within normal limits
Tumor markers	AFP, CEA, and CA19-9 were not elevated	AFP, CEA, and CA19-9 were not elevated
Lesion size	3.3 × 2.1 cm; increased from 2.5 × 1.6 cm over 6 months	1.0 cm
Lesion location	Left medial hepatic segment / segment IV	Left medial hepatic segment / segment IV
Relationship to major vessels	Adjacent to a segmental vessel and the sagittal portion of the left portal vein	Located at a vascular confluence near branches supplying the left medial and left lateral lobes
Main imaging modality	Contrast-enhanced CT	Contrast-enhanced MRI
Main imaging features	Hypervascular nodule with relative hyperdensity on delayed phases; absence of a classic central scar	Round lesion with diffusion restriction, avid arterial enhancement, and slightly high signal on portal/delayed phases
Reason for diagnostic uncertainty	Small lesion without a classic central scar, interval growth, and close proximity to segmental vessels	Complex perivascular location and indeterminate malignant potential
Preoperative differential diagnosis	Atypical FNH, hepatocellular adenoma, and well-differentiated hepatocellular carcinoma	Atypical FNH, hepatocellular adenoma, and well-differentiated hepatocellular carcinoma
Indication for surgery	Documented interval growth, from 2.5 × 1.6 cm to 3.3 × 2.1 cm over 6 months, and persistent diagnostic uncertainty	Complex vascular location and indeterminate malignant potential
ICG protocol	0.5 mg/kg intravenously, 24 h before surgery	0.5 mg/kg intravenously, 24 h before surgery
Imaging system	4K 3D NIR/ICG laparoscopic imaging system, Stellar, OptoMedic Technologies, Foshan, China	4K 3D NIR/ICG laparoscopic imaging system, Stellar, OptoMedic Technologies, Foshan, China
Intraoperative navigation	ICG fluorescence imaging combined with intraoperative ultrasonography	ICG fluorescence imaging combined with intraoperative ultrasonography
Fluorescence pattern	Sharply demarcated, intensely hyperfluorescent lesion	Sharply demarcated, intensely hyperfluorescent lesion
Surgical procedure	Laparoscopic parenchyma-sparing wedge resection	Laparoscopic parenchyma-sparing wedge resection
Operative time	125 min	220 min
Estimated blood loss	50 mL	20 mL
Margin status	Negative surgical margin, 5 mm	Negative surgical margin, 5 mm
Additional fluorescent findings	Several incidental surface punctate hyperfluorescent micro-foci	One additional 0.5 cm punctate hyperfluorescent focus
Pathological diagnosis	Focal nodular hyperplasia	Focal nodular hyperplasia
Pathology of additional fluorescent focus	Not applicable / not resected	Benign nodular hyperplastic change
Resumption of oral intake	Postoperative day 2	Postoperative day 2
Drain removal	Postoperative day 4	Postoperative day 5
Postoperative laboratory recovery	Liver function, biochemical, and coagulation parameters remained within normal limits	Liver function, biochemical, and coagulation parameters remained within normal limits
Postoperative complications	None; no bile leakage, hemorrhage, liver failure, infection, or Clavien-Dindo grade II or higher complications	None; no bile leakage, hemorrhage, liver failure, infection, or Clavien-Dindo grade II or higher complications
Total hospital stay	1 week	10 days
Postoperative day of discharge	POD 4	POD 5
Follow-up duration	8 months	9 months
Follow-up outcome	Asymptomatic; no radiological evidence of residual or recurrent lesion	Asymptomatic; no radiological evidence of residual or recurrent lesion
Patient perspective	Satisfied with the treatment outcome and postoperative recovery; definitive pathological diagnosis helped relieve anxiety	Satisfied with the treatment outcome and postoperative recovery; definitive pathological diagnosis helped relieve anxiety

AFP, alpha-fetoprotein; CA19-9, carbohydrate antigen 19-9; CEA, carcinoembryonic antigen; CT, computed tomography; FNH, focal nodular hyperplasia; ICG, indocyanine green; IOUS, intraoperative ultrasonography; MRI, magnetic resonance imaging; NIR, near-infrared; POD, postoperative day.

**Table 2:** Timeline of clinical course.

Time point	Case 1	Case 2
Initial detection	Incidental left hepatic lesion detected by abdominal ultrasound	1.0 cm lesion detected by screening abdominal ultrasound
Interval assessment	Lesion increased from 2.5 × 1.6 cm to 3.3 × 2.1 cm over 6 months	Not applicable
Preoperative imaging	Contrast-enhanced CT showed a 3.3 × 2.1 cm hypervascular lesion in the left medial hepatic segment	MRI showed a round lesion with diffusion restriction and arterial hyperenhancement in the left medial hepatic segment
Relationship to major vessels	FNH adjacent to the sagittal portion of the left portal vein	Small FNH located at the bifurcation of the portal venous branches to segments II and IV
Diagnostic assessment	Atypical imaging features, interval growth, and proximity to segmental vessels led to persistent diagnostic uncertainty	Complex perivascular location and indeterminate malignant potential supported the decision for surgical treatment
Preoperative laboratory evaluation	Liver function tests, routine biochemical tests, coagulation profile, tumor markers, and viral hepatitis panel were within normal limits	Liver function tests, routine biochemical tests, coagulation profile, tumor markers, and viral hepatitis panel were within normal limits
Preoperative preparation	ICG 0.5 mg/kg was administered intravenously 24 h before surgery	ICG 0.5 mg/kg was administered intravenously 24 h before surgery
Operation	Laparoscopic fluorescence-guided parenchyma-sparing wedge resection with IOUS	Laparoscopic fluorescence-guided parenchyma-sparing wedge resection with IOUS
Intraoperative findings	Strongly fluorescent lesion and several punctate surface hyperfluorescent foci	Strongly fluorescent lesion and one additional punctate hyperfluorescent focus
Pathology	FNH with negative surgical margin, 5 mm	FNH with negative surgical margin, 5 mm; the additional focus showed benign nodular hyperplastic change
Early postoperative course	Oral intake resumed on POD2; drain removed on POD4; no postoperative complications	Oral intake resumed on POD2; drain removed on POD5; no postoperative complications
Discharge	Discharged on POD4 after a total hospital stay of 1 week	Discharged on POD5 after a total hospital stay of 10 days
Follow-up	Asymptomatic at 8 months, with no radiological evidence of residual or recurrent lesion	Asymptomatic at 9 months, with no radiological evidence of residual or recurrent lesion
Patient perspective	The patient was satisfied with the treatment outcome and reported relief after definitive pathological diagnosis	The patient was satisfied with the treatment outcome and reported relief after definitive pathological diagnosis

FNH, focal nodular hyperplasia; ICG, indocyanine green; IOUS, intraoperative ultrasonography; POD, postoperative day.

## Surgical technique and ICG protocol

The following description summarizes the operative strategy used in the two patients.

### ICG administration and timing

A standard dose of 0.5 mg/kg body weight of ICG was administered as a single intravenous bolus approximately 24 h before surgery in both patients. This dose and timing were selected according to our institutional fluorescence-guided liver surgery protocol and previous reports using preoperative ICG for hepatic lesion visualization (5). The rationale was to obtain sufficient lesion-to-background contrast at the time of surgery while limiting excessive background fluorescence.

### Intraoperative imaging platform

All procedures were performed using a high-definition laparoscopic system integrated with near-infrared (NIR) fluorescence capability. Fluorescence imaging was performed with a 4K 3D NIR/ICG laparoscopic imaging system (Stellar, OptoMedic Technologies, Foshan, China). The fluorescent signal was displayed in real time as a green overlay on the standard white-light image. This was complemented by systematic IOUS, which was used to assess lesion depth, map adjacent portal and hepatic venous structures, and plan the transection plane.

## Fluorescence-guided resection strategy

The resection strategy was guided by the combined information obtained from fluorescence imaging and IOUS. After mobilization, a Pringle maneuver tape was pre-positioned for potential intermittent inflow control; however, routine inflow occlusion was not required in these two limited wedge resections. IOUS was used to localize the lesion, define its deep border, and clarify its spatial relationship to adjacent vascular structures. Parenchymal transection was performed with an ultrasonic scalpel, using the visible fluorescence boundary as a superficial landmark and repeated IOUS assessment for deep guidance. Particular attention was paid to preserving fluorescence-negative vascular structures traversing or adjacent to the resection area, consistent with the principle of parenchyma-sparing surgery (6, 7).

When the fluorescent lesion approached a vessel wall, dissection proceeded along the vascular structure under IOUS guidance. Small non-essential branches supplying the lesion could be divided when necessary, whereas major portal venous branches were preserved by careful dissection along the adventitial plane. The minimum microscopic margin was 5 mm in both cases.

## Real-time margin assessment

Fluorescence imaging and IOUS were used together for intraoperative assessment of the transection plane. Fluorescence imaging helped identify whether residual focal hyperfluorescent tissue was present on the superficial cut surface, whereas IOUS was used to assess the deep margin and the relationship between the transection plane and adjacent portal venous branches. In these two cases, the absence of residual focal fluorescence on the transection surface served as an intraoperative adjunctive indicator, and IOUS confirmation supported complete lesion removal while minimizing excision of uninvolved liver parenchyma.

## Discussion

This report describes two cases of radiologically indeterminate focal nodular hyperplasia (FNH) located adjacent to major portal venous branches that were treated by laparoscopic parenchyma-sparing excision with preoperative indocyanine green (ICG) administration, real-time fluorescence imaging, and intraoperative ultrasonography (IOUS). In both cases, fluorescence imaging assisted superficial lesion localization, whereas IOUS provided depth assessment and vascular mapping. This complementary strategy enabled complete excision while preserving the adjacent portal venous branches.

The main technical challenge in these cases was not the extent of parenchymal transection but the need for precise dissection around the major portal venous branches. Fluorescence alone could not define deep margins or vascular anatomy due to its limited tissue penetration (typically 5–10 mm) (8). Therefore, IOUS was indispensable for identifying the deep border of the lesion and mapping adjacent portal branches. The practical value of fluorescence in this setting was to provide a surface landmark that could be continuously correlated with IOUS findings during transection.

We acknowledge that ICG dosing and timing remain heterogeneous across studies, and that our institutional protocol (0.5 mg/kg, 24 h before surgery) represents one of several acceptable strategies (5). Shorter preoperative intervals may be associated with increased background fluorescence or false-positive signals, whereas longer intervals may reduce lesion-to-background contrast due to ICG washout (6). Our choice of a 24-h interval was intended to balance sufficient lesion retention with acceptable background signal, consistent with previous reports using preoperative ICG for hepatic lesion visualization (6). Nevertheless, the optimal dose and timing for perivascular FNH specifically remain to be defined, and our findings should be interpreted with this protocol in mind.

Li et al. (4) previously reported a robotic series of 23 ICG-guided FNH resections, demonstrating the feasibility of fluorescence navigation for this benign lesion. The present report differs from that experience in two important respects. First, our cases involved smaller lesions (3.3 cm and 1.0 cm) with atypical imaging features that precluded a definitive non-invasive diagnosis. Second, the lesions were located in close proximity to major portal venous branches, a perivascular setting where standard anatomical resection or wedge excision without image guidance carries an elevated risk of vascular injury. While Li et al. focused on the general utility of ICG for FNH localization, the present report describes a specific complementary role for ICG fluorescence when combined with IOUS to assist vascular preservation and parenchyma-sparing excision in this challenging anatomical context.

Current guidelines favor conservative management for typical FNH, particularly in asymptomatic patients with diagnostic imaging findings (1). The Italian guidelines specifically recommend surveillance only in symptomatic patients or in lesions that are large, subcapsular, or peri-caval (2). However, individualized decision-making may be warranted in cases with atypical imaging features, documented interval growth, diagnostic uncertainty, or a complex perivascular location requiring parenchyma-sparing consideration. In Case 1, the lesion showed documented interval growth on imaging. In Case 2, atypical imaging features precluded a definitive non-invasive diagnosis of FNH even after multidisciplinary team (MDT) discussion. In both instances, the patients actively opted for surgery after informed consent. Thus, while the Italian guidelines provide a valuable framework, their lower recommendation level for FNH management acknowledges that individualization is appropriate when atypical features, diagnostic uncertainty, perivascular anatomy, or patient preference intervene. The indications for surgery in the present cases were based on persistent diagnostic uncertainty, perivascular location, technical feasibility of parenchyma sparing excision, and patient preference after informed consent.

It is important to note that both patients in this report had non-cirrhotic, non-steatotic livers. In cirrhotic or steatotic parenchyma, ICG kinetics may be significantly altered due to impaired hepatocellular uptake, cholestasis, or altered perfusion, potentially reducing lesion-to-background contrast (8). Conversely, in diseased or cirrhotic livers, non-cancerous nodular lesions may show ICG fluorescence retention and produce false-positive findings, thereby complicating intraoperative interpretation (9). Therefore, while our findings support ICG guidance in healthy livers, extrapolation to patients with chronic liver disease requires caution and further study.

In both cases, fluorescence imaging provided visible contrast between the lesion and surrounding parenchyma that assisted in defining the superficial resection plane. This fluorescence pattern aligns with prior descriptions of FNH and FNH-like lesions, which typically exhibit homogeneous hyperfluorescence relative to surrounding liver parenchyma (4, 10). Nevertheless, ICG fluorescence functioned as an intraoperative navigation adjunct that needed to be interpreted together with preoperative imaging, IOUS, and histopathology, rather than as a lesion-specific diagnostic test.

An additional observation was the presence of punctate hyperfluorescent foci on the liver surface in both patients. One focus resected in Case 2 showed benign nodular hyperplastic change, whereas the multiple foci observed in Case 1 were not resected. These findings should be interpreted cautiously because ICG retention is not specific for FNH. False-positive ICG fluorescence has been reported in benign, regenerative, hyperplastic, cholestatic, steatotic, and treatment-related hepatic lesions, with false-positive rates approaching 40%−50% in some series (9, 11–13). Quantitative fluorescence assessment, such as tumor-to-background ratio, may help distinguish true lesions from false-positive nodules (9, 11–13). Given the small sample size of this two-case report, no conclusion can be drawn regarding the nature of the unresected foci or the diagnostic significance of this observation. The clinical relevance of such punctate fluorescence remains uncertain and requires longer follow-up and systematic evaluation.

## Conclusion

In these two selected cases, the integration of preoperative ICG with laparoscopic IOUS enabled real-time visual and mapping guidance that facilitated precise, negative-margin, parenchyma-sparing resection of perivascular focal nodular hyperplasia (FNH). The incidental punctate hyperfluorescent foci observed intraoperatively should be interpreted with caution, as their clinical significance remains uncertain and warrants further evaluation with long-term follow-up or histologic correlation. These findings support further investigation in larger prospective studies to define the reproducibility, optimal patient selection, and clinical value of this dual-modality approach.

## Limitations

This report has several limitations. First, this is a retrospective, descriptive report of only two cases. Second, both patients had non-cirrhotic, non-steatotic livers; therefore, the findings should not be extrapolated to patients with chronic liver disease. In such patients, altered ICG kinetics and potentially higher false-positive fluorescence rates may reduce lesion-to-background contrast. Third, the follow-up duration was limited to 8–9 months, which is insufficient to assess the long-term natural history of the incidental punctate hyperfluorescent foci. Fourth, no quantitative fluorescence metric, such as lesion-to-background ratio, was measured. Finally, only one of the multiple punctate hyperfluorescent foci observed in the two cases was histologically examined, precluding any inference regarding the nature or clinical significance of the remaining foci.

## CARE statement

This case report was prepared in accordance with the CARE guidelines (14). Key clinical information and timelines are summarized in Tables 1, 2.

## Data Availability

The original contributions presented in the study are included in the article/supplementary material, further inquiries can be directed to the corresponding author.
